# Impact of Acidic Corrosion on the Stability of Dented Thin-Walled Cylindrical Steel Tanks

**DOI:** 10.1021/acsomega.4c10839

**Published:** 2025-02-24

**Authors:** Mahyar Maali, Dilek Biten, Yasin Tizi, Abdulkadir Cüneyt Aydın

**Affiliations:** 1 Faculty of Engineering and Architecture, Civil Engineering Department, Erzurum Technical University, Erzurum 25050, Turkey; 2 Maali Çelik Ar−Ge Danışmanlık Müh. İnş. Taah. Tarım ve Hayvancılık Company, Atateknokent, Erzurum 25030, Turkey; 3 Engineering Faculty, Department of Civil Engineering, Atatürk University, Erzurum 25030, Turkey; 4 Academy Sağlık Hiz. Müh. İnş. Taah. Elekt. Yay. Company, Atateknokent, Erzurum 25030, Turkey

## Abstract

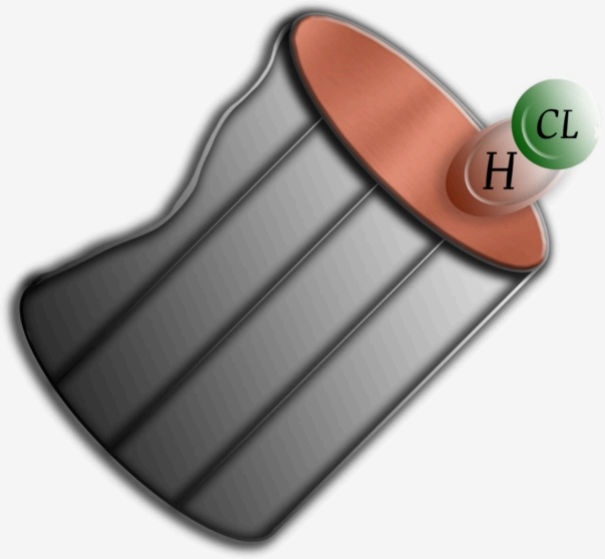

Thin-walled steel structures are renowned for their high strength-to-weight ratio; however, they are particularly prone to degradation from mechanical imperfections and chemical processes. The limited thickness of these structures amplifies their vulnerability to corrosion, especially in acidic environments. This study examines the synergistic effects of mechanical dents and chemical corrosion on the buckling capacity of thin-walled cylindrical steel shells made from 0.45 mm-thick galvanized steel sheets. To maintain structural realism, an r/t ratio of 445 was achieved through precise cutting, while dents of varying sizes (t, 2t, 3t), where t represents the shell’s thickness, were introduced to simulate mechanical imperfections. The chemical interactions were rigorously investigated, focusing on the microstructural changes triggered by 2.5 and 5% HCl solutions, which led to oxidation, material loss, and subsequent reductions in mechanical stability. Weight loss measurements confirmed the material degradation, with corrosion effects correlating to increased dent sizes, further exacerbating structural vulnerability. The findings revealed that both dent severity and corrosion level significantly influenced the buckling capacity, demonstrating the critical interplay between mechanical and chemical factors. This study provides insights into the degradation mechanisms in thin-walled steel structures, offering a foundation for improved material resilience and corrosion mitigation strategies in engineering applications.

## 1 Introduction

Steel is commonly used in industrial applications due to its high strength-to-weight ratio and durability. Thin-walled cylindrical steel tanks, known for their economic and structural efficiency, are widely utilized in storage, transport, and manufacturing. However, these tanks are vulnerable to detrimental effects like dents and corrosion, which weaken their load-bearing capacities. Dents create stress concentrations, while corrosion increases the risk of material loss and deformation.

One of the primary advantages of thin-walled steel structures is their ability to bear substantial loads while utilizing less material compared to traditional thick-walled counterparts. Cold-formed thin-walled steel, for instance, is manufactured by bending thin steel plates into various shapes, allowing for efficient load-bearing capabilities without increasing the section area.^1^ This method not only enhances material efficiency but also contributes to the overall sustainability of construction practices by minimizing waste.^2^ However, the design of these structures must account for potential local buckling, which can occur under axial loads, shear, or bending due to their thin walls.^3^ The research has shown that optimizing the design of these structures can significantly improve their stability and performance under various loading conditions.^4,5^

The buckling behavior of thin-walled steel tanks remains a key research topic. The consequences of buckling in steel tanks can be analyzed through various dimensions, including material degradation, environmental repercussions, implications for human health, risks associated with occupational safety, and potential legal liabilities.^6^ These tanks, vital for liquid storage in industries such as petroleum and energy, face challenges related to design, manufacturing defects, and environmental impacts. Maintaining their structural integrity is essential, as buckling is influenced by factors like geometric imperfections, material degradation, and external pressures such as wind or seismic activity. An important factor affecting the buckling behavior of these tanks is the presence of dents. Dents can introduce geometric imperfections that significantly reduce the buckling capacity of thin-walled structures. They can occur due to manufacturing defects, errors during assembly, or damage during operational processes. Such localized geometric imperfections can severely impact the structural integrity of the tank and adversely change its buckling behavior. In axially compressed cylindrical steel shells, these imperfections can lead to critical buckling modes that differ from those observed in undamaged structures.^7^ Additionally, the buckling behavior of cylindrical tanks highlights the necessity of considering dents in the design and evaluation process.^8^

Corrosion is another critical factor that threatens the integrity of thin-walled steel tanks. Corrosion can occur in various forms depending on the materials and environments involved. It leads to the weakening of the tank material over time and consequently reduces its load-bearing capacity. The spread of corrosion on the tank surface, in addition to localized defects and dents, is an important factor affecting the overall strength of the tank. Particularly, when exposed to wind and vacuum pressures, corrosion combined with other stress factors such as wind loads can further increase the vulnerability of steel tanks to buckling.^9^ Additionally, corrosion not only alters material properties but also changes the buckling behavior under external pressures; hence, regular maintenance of steel tanks is crucial for mitigating corrosion-related risks.^10^ The buckling behavior of these tanks is also influenced by external environmental factors. Due to their thin-walled nature, cylindrical steel tanks are susceptible to buckling under wind loads.^11^ These studies collectively demonstrate the multifaceted nature of buckling behavior in thin-walled tanks, necessitating a comprehensive design and analysis approach that takes into account various loading conditions and material degradations.

Research has shown that geometric imperfections such as dents, significantly affect the buckling behavior and stress distribution in thin-walled structures. The dents can lead to a reduction in the buckling capacity of cylindrical shells, with factors such as dent depth and location playing a role.^12,13^ Experimental studies have revealed that the presence of dents notably decreases the initial buckling loads compared to flawless shells.^13^ Stress concentrations in dented regions are significantly higher and can be measured through mathematical models that relate dent dimensions to stress concentration factors.^14^ The interaction between intentional and unintentional imperfections complicates the evaluation of buckling strength and necessitates advanced finite element analyses for accurate prediction of structural behaviors.^15^

Experimental investigations have provided valuable insights into how dent characteristics affect buckling capacity. Korucuk et al.^16^ conducted experiments that demonstrated how variations in dent size and shape directly impact the buckling strength of thin-walled cylindrical shells. Similarly, Prabu et al.^17^ explored the effects of dent dimensions on the buckling behavior of short cylindrical shells, revealing that both the extent and depth of the dent significantly influence the critical buckling load.

Theoretical analyses complement experimental findings, providing a deeper understanding of the mechanics involved. The study of Ghazijahani et al.^18^ on corrugated thin cylindrical shells under uniform external pressure illustrates the complex interactions between geometric imperfections and buckling behavior. Moreover, the impact of dent parameters on the critical buckling load has been systematically analyzed. Modifying dent characteristics can potentially improve the buckling performance of thin-walled steel cylinders.^19^

Maraveas et al.^20^ compared the buckling resistance of two large-diameter, thin-walled steel tanks under wind loads using numerical methods from Eurocodes and analytical formulations from design standards. They found that EN1993–1–6^21^ imperfection amplitudes are overly conservative, and API 650^22^ methods lack precision in quantifying buckling. They provided recommendations on boundary conditions and imperfection modeling to enhance design accuracy. Badamchi and Showkati^23^ investigated the buckling behavior of thin-walled steel pipes under combined axial compression and external pressure through experimental, numerical, and analytical methods. They found that axial precompression significantly reduced the buckling load and altered the failure modes, forming circumferential waves under external pressure. Their modified analytical formula accurately predicted buckling loads, aligning well with experimental results. Subramanian et al.^24^ investigated oxygen corrosion in heat exchanger tubes used in a steam generation system through experimental techniques, including remote field eddy current testing, microscopy, and chemical analysis. They found that dissolved oxygen in boiler feedwater was the primary cause of corrosion, with additional contributions from chlorine, sulfur, and inadequate corrosion inhibitors. They proposed measures such as oxygen scavengers, deaeration systems, and improved boiler feedwater monitoring to prevent further failures. Ren et al.^25^ reviewed recent advancements in stiffness enhancement methods for thin-walled aerospace structures, focusing on both passive techniques (e.g., laminate optimization and reinforcement ribs) and active approaches using smart materials like shape memory alloys and piezoelectrics. They identified that passive methods are widely applied but increase weight, while active methods offer promising potential despite current limitations in practical applications. They highlighted the need for future research to bridge the gap between intelligent material studies and engineering applications. Saifi et al.^26^ investigated the dynamic response of a thin-walled cylindrical water tank under blast loading using numerical simulations with advanced computational methods. They found that the presence of water significantly reduced deformation and damage, enhancing the tank’s resilience to explosions due to the “added mass” effect. They concluded that filling tanks with water can effectively mitigate damage from chemical explosive detonations. Zhang et al.^27^ developed a theoretical model to calculate the critical buckling pressure of thin-walled metal liners in composite overwrapped pressure vessels and validated it through experiments. They found that the liner material and thickness significantly influence the pressure ratio, with higher-yield-strength alloys achieving better fiber layer utilization. They concluded that considering liner buckling can enhance composite overwrapped pressure vessels design accuracy, reduce testing costs, and improve safety. Attia et al.^28^ developed a novel finite element formulation to predict the nonlinear response of conical shells under various loading conditions using Love-Kirchhoff shell theory and Fourier series. They found the method to be highly accurate and computationally efficient, requiring fewer degrees of freedom compared to conventional models. They observed distinct stress and displacement patterns depending on the loading type and shell geometry.

**Figure 1:**
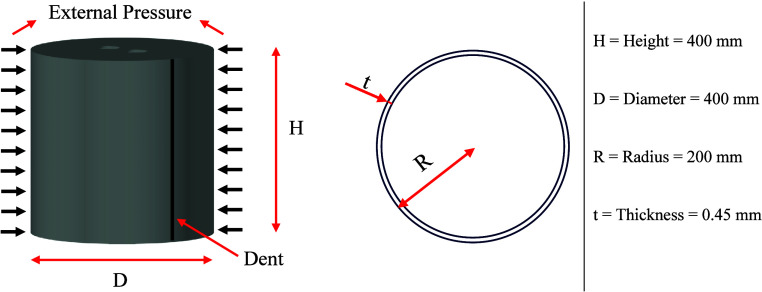
Overview of experimental test specimen.

These structural weaknesses in steel tanks are considered to have significant implications for safety and environmental impacts. Therefore, detailed investigation of the effects of dents and corrosion is crucial for the service life and safe use of tanks. This study uniquely contributes to the existing body of knowledge by addressing the synergistic interaction between geometric imperfections and corrosion-induced chemical degradation, a topic insufficiently explored in prior research. By simulating realistic operational conditions involving external pressures and chemical exposure, this research provides critical insights into how these coupled effects influence the mechanical performance of thin-walled cylindrical steel tanks. The findings not only fill existing gaps in the literature but also offer practical guidance for the design, maintenance, and safety assessment of such structures. Building upon this foundational gap, the buckling capacity of thin-walled cylindrical steel specimens (H = 400 mm, D = 400 mm, R = 200 mm, t = 0.45 mm) was experimentally investigated under external pressure (Figure 1.). The specimens included three dented configurations (1t, 2t, and 3t) with variations introduced through exposure to 2.5% and 5% hydrochloric acid solutions to simulate corrosion effects. To assess the impact of dents and corrosion, the results were compared with those of dent-free specimens from a previous study in the literature.^6^ The investigation highlighted how these coupled effects degrade the steel’s microstructure, reduce its load-bearing capacity, and alter its structural response under external pressures, providing a deeper understanding of the performance of steel tanks under realistic operational conditions.

## 2 Experimental Study

### 2.1 Details of Specimens

**Figure 2:**
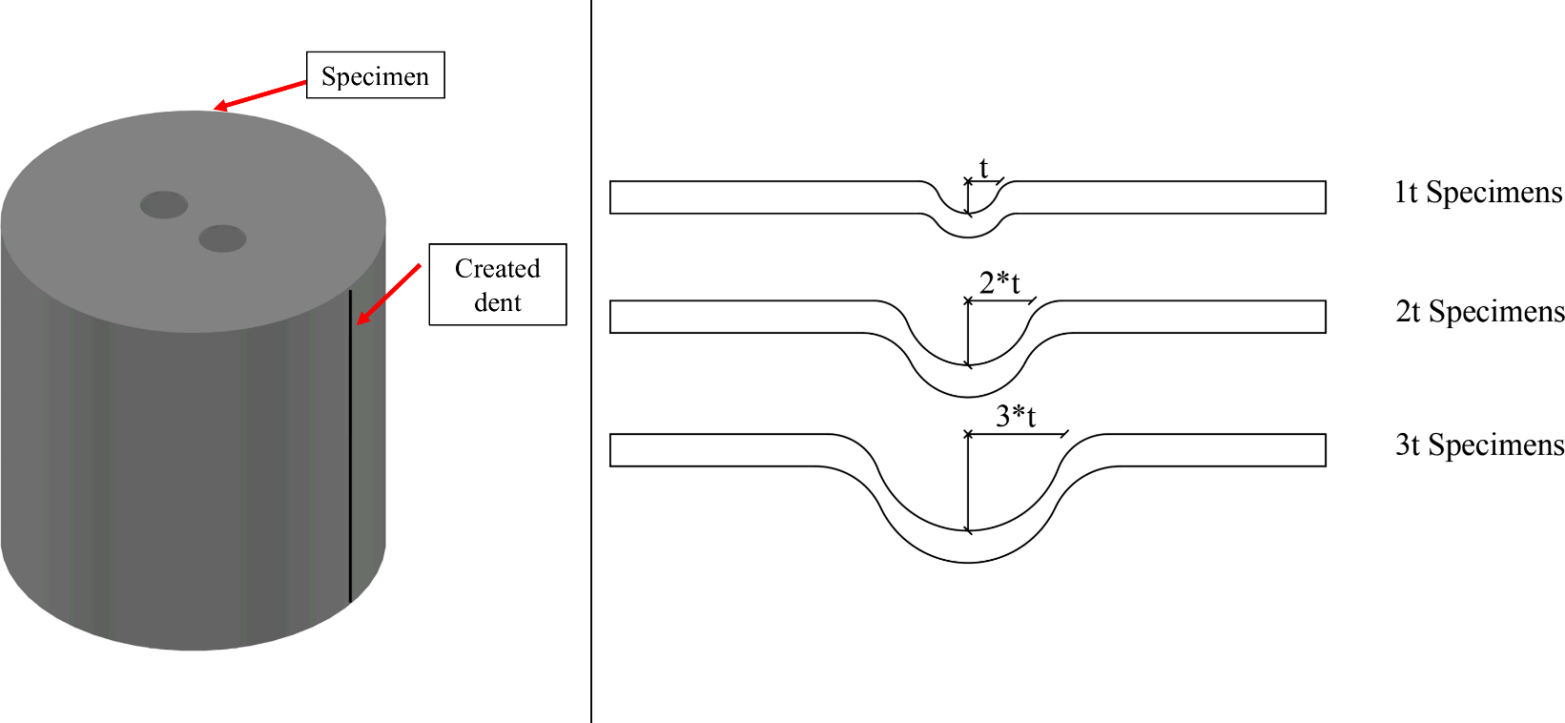
Dent sizes for dented specimens.

The details of the specimens are presented in Table 1. Steel sheets with a thickness of 0.45 mm were utilized to fabricate the cylindrical steel shell specimens. To ensure the specimens demonstrated realistic behavior, the radius-to-thickness (r/t) ratios were chosen to range between 300 and 1000.^29^ Each cylindrical shell has a radius of 200 mm, a height of 400 mm, and an r/t ratio of 445, which was within the desired range for realistic testing conditions. The steel sheets were precision-cut to the required dimensions using CNC machining and then formed into cylindrical shapes through soldering. Each cylinder contains a single dent in the form of a vertical line extending from the top to the bottom, ensuring consistent placement and orientation across all specimens (Figure 2.). Steel plates, also with a radius of 200 mm, were used as covers, each having two perforations. One hole served to connect a vacuum pump, while the other was fitted with a load cell. A total of nine cylindrical shell specimens were prepared, consisting of three noncorroded specimens and six corroded specimens. Among the corroded samples, three were exposed to a 2.5% HCl solution, and the remaining three to a 5% HCl solution. The weight loss measurements were taken twice for each corroded specimen: once before corrosion and once after the corrosion process. These measurements were recorded using a precise balance to ensure accuracy. The weight loss values for all specimens are presented in Table 1.

**Table 1:** Properties of the Experimental Specimens

			dimensions (mm)			weight of the specimens (mg × 10^4^)		
group	specimen	HCl ratio (%)	**thickness**	**height**	**radius**	**before corrosion**	**after corrosion**	weight loss ratio (%)
noncorroded	P-1t		0.45	400	200			
	P-2t							
	P-3t							
corroded	2.5%-1t	2.5				155	144.6	6.7
	2.5%-2t					155	144	7.1
	2.5%-3t					154.5	143.9	6.8
	5%-1t	5				155	140.7	9.2
	5%-2t					154.5	139.4	9.8
	5%-3t					155	140.3	9.5

The dent regions for each group of specimens—noncorroded, 2.5% corroded, and 5% corroded—were modified in a stepwise manner. The terms 1t, 2t, and 3t in the specimen names refer to the radius of the dent being examined in the specimen. Here, the value of t represents the thickness of the cylinder (0.45 mm), and the dent sizes were created at values corresponding to the thickness, twice the thickness, and three times the thickness, respectively (Figure 2).

The cylindrical vessels were manufactured using DX51D steel, a hot-dip galvanized low-carbon steel categorized under the EN 10346:2015 standard,^30^ which specifies technical delivery conditions for continuously hot-dip coated steel flat products intended for cold forming. The chemical composition of DX51D steel typically includes a maximum carbon content of 0.12%, manganese content up to 0.6%, phosphorus content up to 0.1%, sulfur content up to 0.045%, and aluminum content ranging from 0.015% to 0.15%. The tensile properties presented in Table 2, including a Young’s modulus of 210.01 GPa, a yield stress of 198.82 MPa, an ultimate stress of 342.44 MPa, and a Poisson’s ratio of 0.29, were obtained through tensile testing of coupon samples conducted in accordance with ASTM E8m.^31^ These values align with the mechanical behavior expected for DX51D steel.

**Table 2:** Tensile Properties of Cylindrical Shell

Young’s modulus (GPa)	yielding stress (MPa)	ultimate stress (MPa)	Poisson’s ratio
210.01	198.82	342.44	0.29

**Figure 3:**
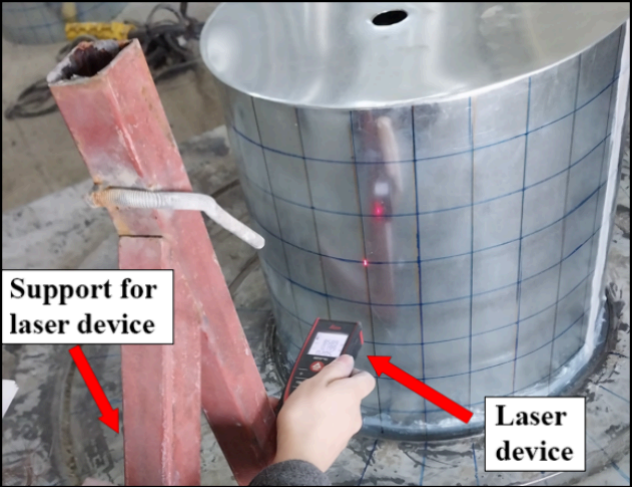
Setup for measuring imperfection values.

**Figure 4:**
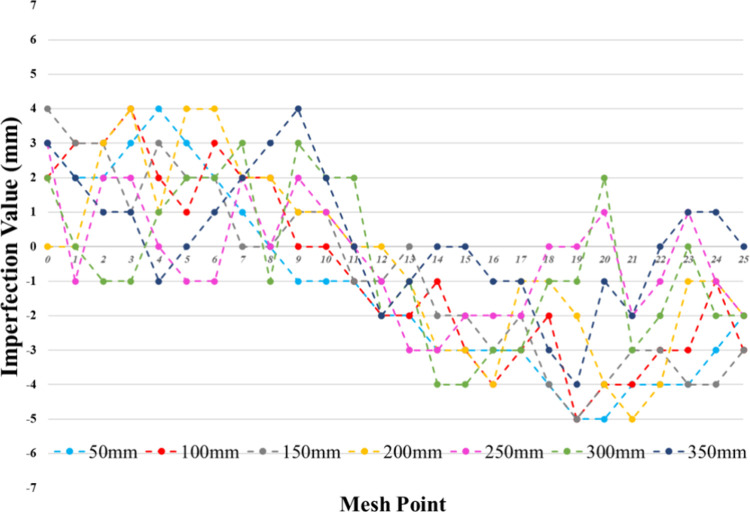
Measured geometric imperfections.

In the experimental setup, geometric imperfections of all cylindrical specimens were measured prior to testing using a precise laser measurement device, as shown in Figure 3. Measurements were conducted at predefined mesh points along the specimen heights of 50, 100, 150, 200, 250, 300, and 350 mm. Figure 4 presents the maximum irregularities at these mesh points, which were determined to be 5 mm, with all specimens generally exhibiting imperfection values approximately in the range of 3–5 mm. These irregularities were compared to similar studies from literature, which indicate that irregularities below this range are typically considered acceptable for thin-walled cylindrical structures.^6,32^ Furthermore, during testing, no unexpected deformation patterns or abnormal load-bearing behaviors were observed that could be attributed to these initial irregularities, as the failure modes were concentrated in the connection and dent regions of the cylinders, which aligned with expectations. This consistency in experimental results across all specimens reinforces the assumption that the recorded geometric deviations had a negligible impact on the test outcomes.

For the cylindrical shell specimens to be subjected to corrosion, solutions of 2.5% HCl and 5% HCl were used. The corrosion behavior of metals in acidic environments is another critical area of research involving hydrochloric acid. Studies about corrosion behavior of metals in acidic environments have shown that HCl can significantly accelerate the corrosion rates of various metals.^33−35^ The choice of using 2.5% and 5% acid solutions is based on their common application in the literature to represent varying degrees of acidic effects for different research purposes.^6,34−37^ The investigation of the corrosion effects of hydrochloric acid solutions on thin-walled shells is critical for understanding material degradation in various engineering applications. The use of HCl solutions can significantly impact the structural integrity of thin-walled cylindrical shells, primarily due to the acid’s aggressive nature toward metals. Specifically, the 2.5% and 5% acidic solutions were selected as they align with real-world scenarios, such as industrial cleaning processes, exposure to acidic rain in polluted regions, or storage tanks containing diluted acidic solutions.^38,39^ These concentrations allow for the simulation of mild to moderate corrosion effects observed in practical conditions without causing excessive degradation. Furthermore, previous studies have demonstrated that these levels are effective in inducing measurable corrosion while avoiding rapid or extreme material failure, thus providing reliable experimental conditions for evaluating material behavior.^6,37,40^ In this study, the hydrochloric acid used was produced in accordance with the TS-EN ISO 9001:2008 standard.^41^ The purity ranged between 30 and 32%, and the density was within the range of 1.15–1.16 g/cm^3^. The iron (Fe) content was 0.0005%, and the arsenic (As) content was 0.0001%. To assess the extent of corrosion, the weight loss method was applied. Corroded specimens were exposed to the acid for 24 h and subsequently subjected to buckling tests. This approach allowed for a comparative analysis of the effects of dent and corrosion, both individually and in combination, on the buckling capacity.

### 2.2 Details of the Test Setup

**Figure 5:**
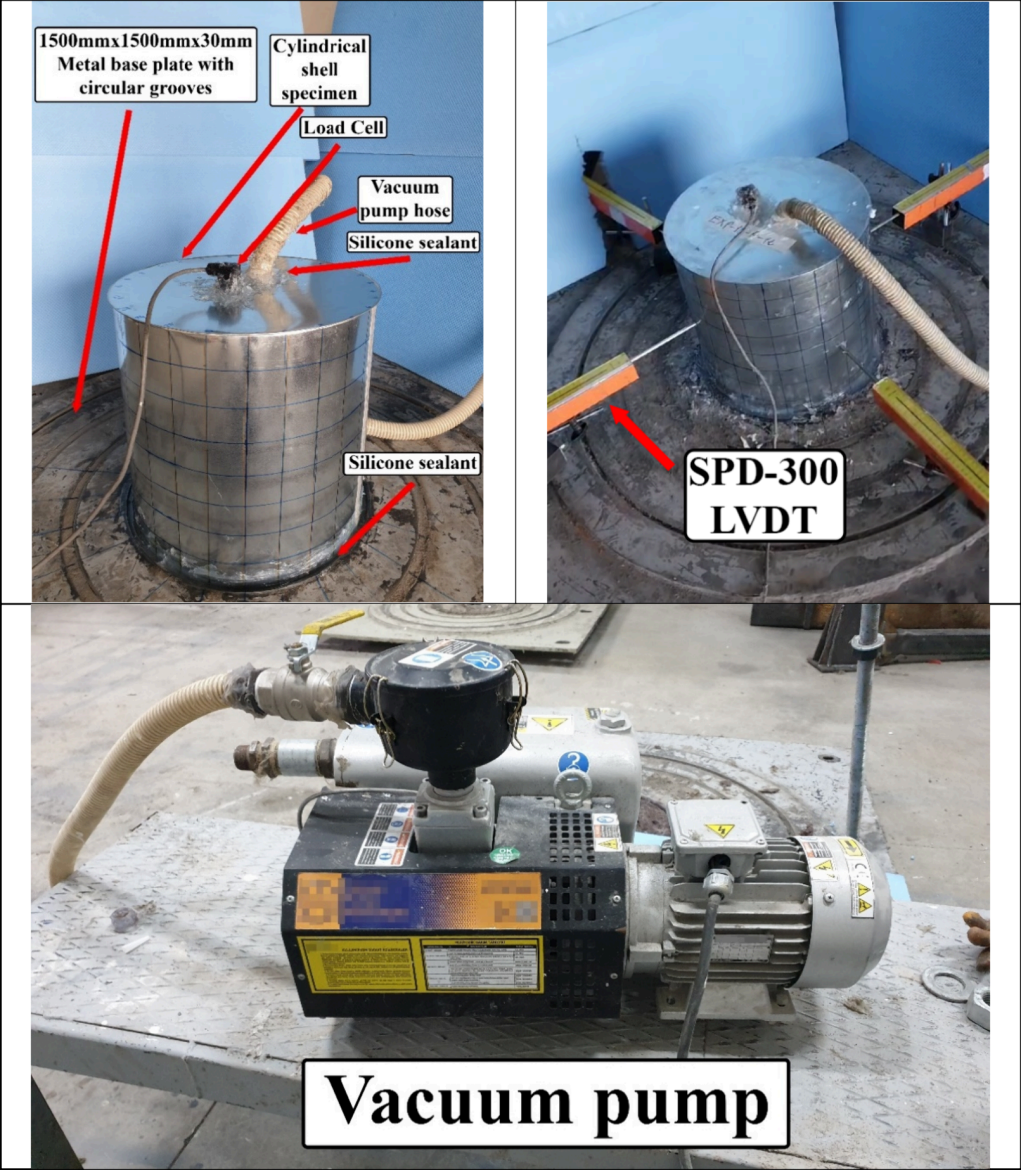
Test setup for experimenting cylindrical shells.

The experimental setup is shown in Figure 5. The setup includes a cylindrical specimen positioned on a metal plate with circular grooves using cold silicone to prevent air leakage. One of the holes on the cylindrical specimen is connected to a vacuum pump pipe with an external pressure capacity of up to 6 bar, while a load cell that measures the pressure force in the specimen instantaneously is placed in the other hole. Horizontal deformations resulting from the axial pressure were measured instantaneously using LVDTs (Linear Variable Differential Transformers). Four LVDTs were positioned at equal intervals around the cylindrical specimen.

## 3 Results and Discussions

### 3.1 Buckling Response

**Figure 6:**
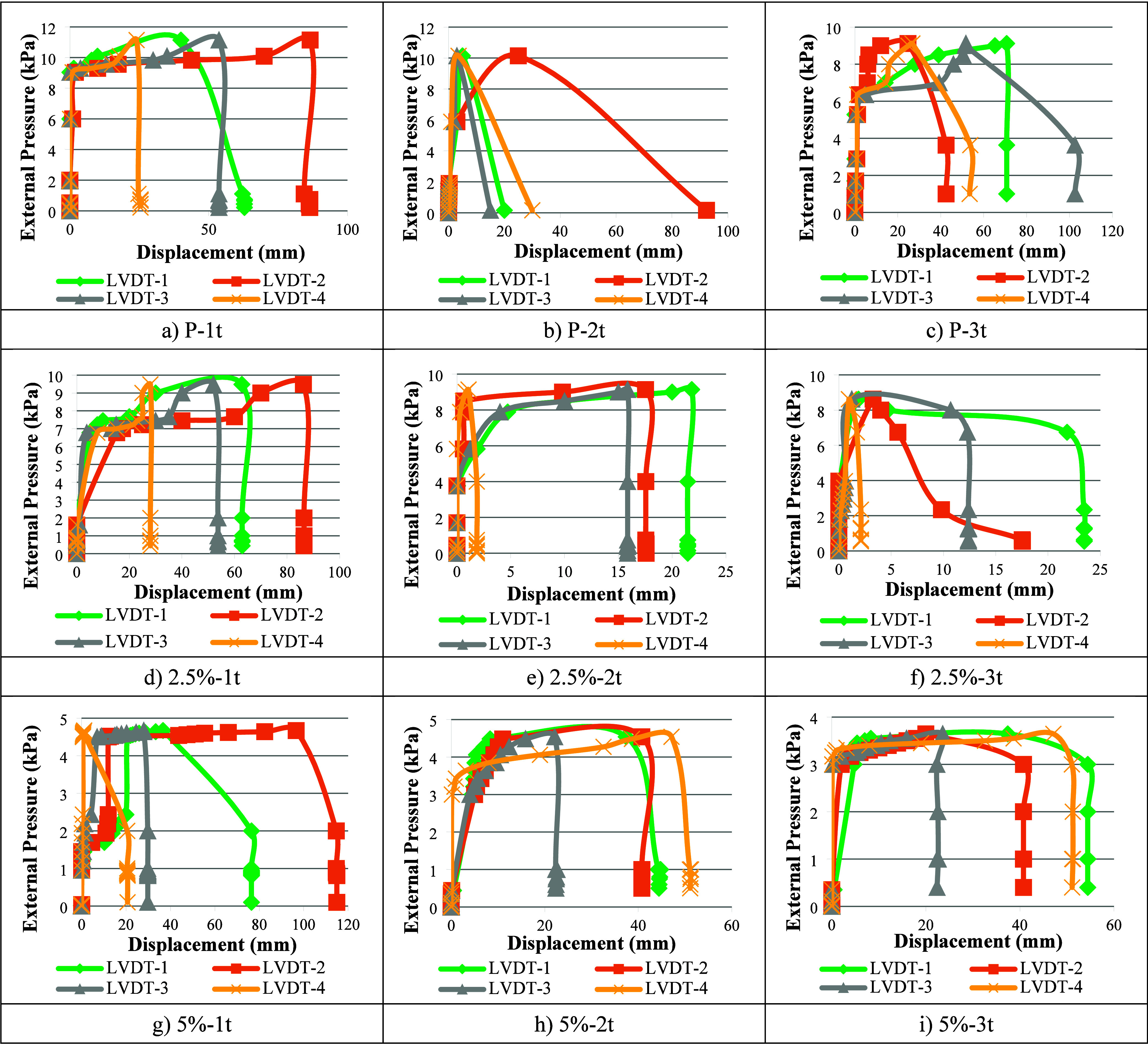
External pressure vs displacement graphs of the specimens.

The buckling values of thin-walled steel specimens in the Table 3. is presented under the influence of both mechanical dents (1t, 2t, 3t) and corrosion effects (%2.5 and %5 HCl solutions). The overall buckling load-to-initial buckling load ratios range from 1.04 to 4.74, while the collapse load-to-overall buckling load ratios vary between 1.04 and 2.43. The initial buckling value is defined as the value recorded when the first movement is detected from the measurement points. General buckling is defined as the first moment when all LVDTs begin to simultaneously measure values, and collapse buckling is expressed as the maximum load the specimen can withstand before failure. The external pressure - displacement graphs obtained from the LVDTs for each specimen are shown in Figure 6.

**Table 3:** Initial Buckling Load, Overall Buckling Load, and Collapse Load of Samples

group	specimen	heights of LVDTs (mm)	initial buckling load (kPa)	overall buckling load (kPa)	collapse load (kPa)	overall buckling load-to-initial buckling load	collapse load-to-overall buckling load	total buckling waves
non-corroded	P-1t	200	2.01	9.04	11.14	4.50	1.23	7
	P-2t		1.89	5.87	10.13	3.11	1.73	2
	P-3t		1.70	5.28	9.12	3.11	1.73	6
corroded	2.5%-1t		1.61	6.78	9.48	4.22	1.40	6
	2.5%-2t		1.70	3.76	9.14	2.21	2.43	7
	2.5%-3t		1.79	3.96	8.62	2.21	2.18	6
	5%-1t		0.95	4.50	4.67	4.74	1.04	7
	5%-2t		0.44	3.07	4.54	6.98	1.48	7
	5%-3t		0.35	3.17	3.65	9.01	1.15	6

It has been observed that the dents, corrosion, and imperfections in the specimens have a reducing effect on the buckling capacity, while increasing the number of waves. This is due to the fact that these effects reduce the structural integrity, leading to earlier buckling and a higher wave formation in the specimens.

### 3.2 Effect of Dent on the Buckling Behavior of Cylindrical Specimens

**Figure 7:**
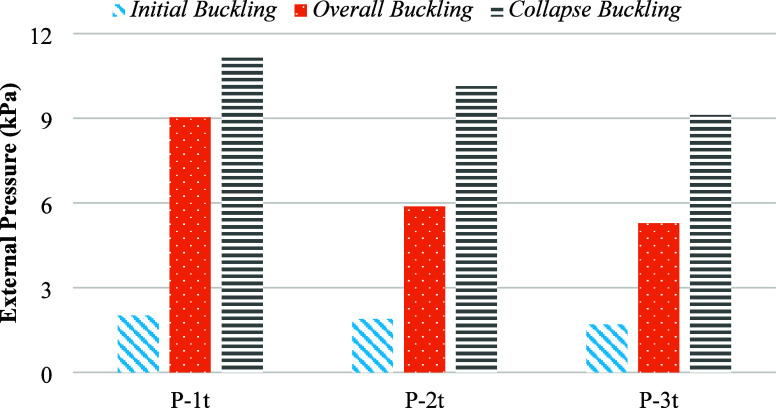
Effect of dent on buckling capacity.

Table 3 indicates that as the dent thickness increases from 1t to 3t, the buckling performance of the cylindrical specimens generally decreases. For both noncorroded and corroded specimens, the initial buckling load, overall buckling load, and collapse load values are reduced with increasing dent thickness. This trend demonstrates that larger dent thicknesses adversely affect the load-bearing capacity and stability of the specimens, leading to lower buckling and collapse loads. The effect of increasing dent thickness on the initial, overall, and collapse buckling loads of noncorroded specimens is illustrated in Figure 7.

### 3.3 Effect of Corrosion-Induced Degradation on Material Loss and the Buckling Behavior of Cylindrical Specimens

**Figure 8:**
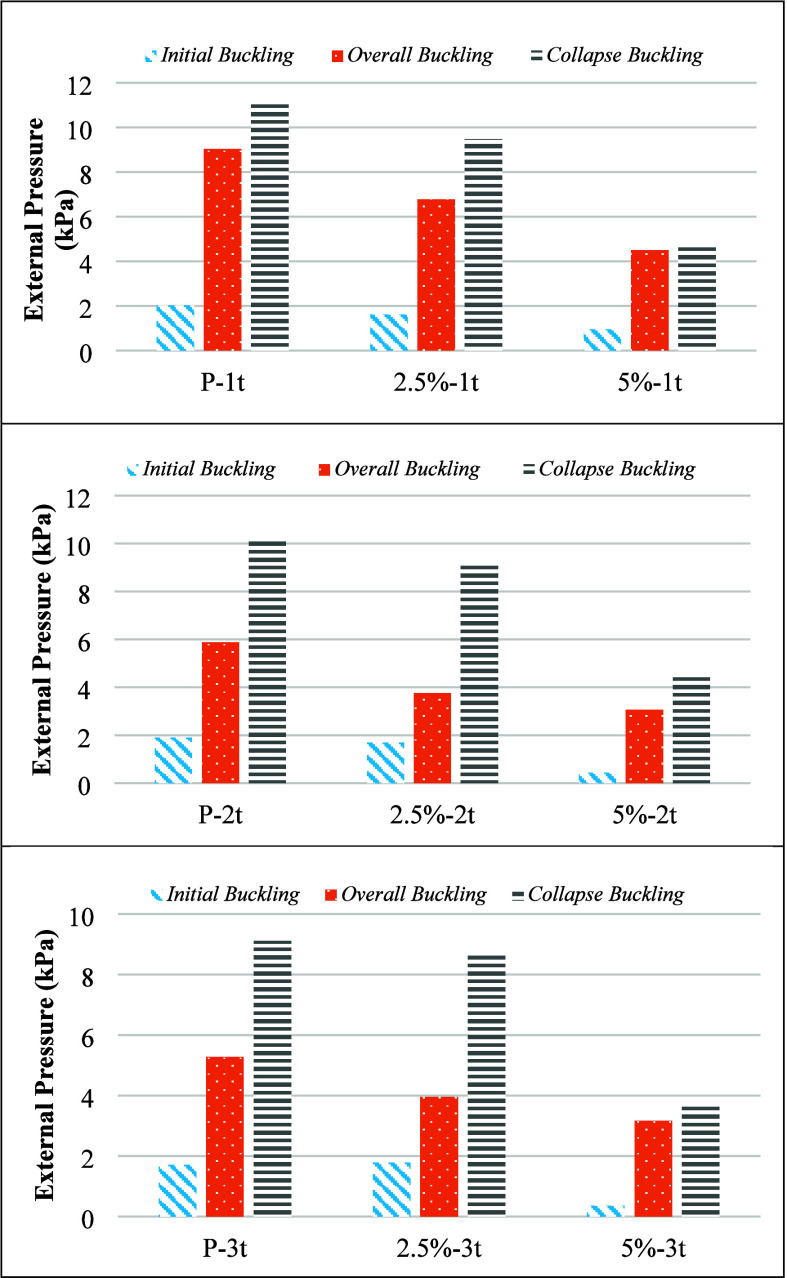
Effect of corrosion on buckling capacity.

Table 3. reveals that the severity of corrosion has a direct impact on the buckling behavior of cylindrical specimens, with higher corrosion levels resulting in more pronounced reductions in buckling and collapse loads. While both 2.5% and 5% corrosion levels negatively affect the structural performance, the effects are more severe at 5% corrosion, leading to significantly lower initial, overall, and collapse buckling loads. Furthermore, the ratios of load parameters and the number of buckling waves are also influenced more drastically by the higher corrosion level, indicating greater structural degradation and instability. The variation in initial, overall, and collapse buckling loads with the increasing percentage of solution exposure in the specimens is illustrated in Figure 8.

The weight loss for 1t, 2t, and 3t dented specimens ranged between 6.7–7.1% in 2.5% HCl solution and 9.2–9.8% in 5% HCl solution, with 2t specimens showing slightly higher values in both cases (Table 1). This observation may be attributed to the specific geometry of the 2t dent, which likely facilitated better circulation and interaction of the acidic solution within the dent region. In contrast, the deeper geometry of the 3t dents could have restricted acid flow, creating stagnant zones that slowed down the corrosion process. Additionally, the stress distribution around the 2t dent might have promoted microcrack formation and propagation, accelerating the material’s degradation. Variations in surface uniformity or microstructural characteristics among specimens could also have contributed to this observation. Further investigation into the fluid dynamics within the dent regions and the stress distribution at different dent depths could provide additional insights into these trends.

**Figure 9:**
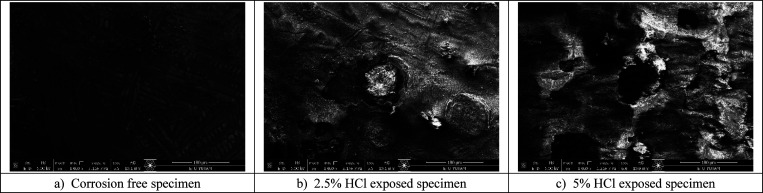
SEM images for specimens.

The SEM images obtained from the E-SEM analysis are presented in Figure 9, providing a visual representation of the corrosion progression in the specimens.

In Figure 9, SEM images illustrate the progression of pitting corrosion in steel specimens exposed to hydrochloric acid solutions, highlighting the impact of increasing acid concentration on surface degradation. The first specimen, which was not exposed to HCl, exhibits a relatively smooth surface with visible machining marks and no apparent signs of corrosion damage. The microstructure appears uniform, with no evidence of localized attack, indicating the material’s initial integrity before acid exposure. In contrast, the second specimen, which was subjected to a 2.5% HCl solution, shows the early stages of pitting corrosion. Small pits and depressions are visible across the surface, indicating localized material loss. Additionally, the surface appears roughened due to the initiation of corrosion activity, likely caused by the interaction between the acidic environment and the steel’s microstructural heterogeneities. The presence of these pits suggests that chloride ions (Cl^–^) have begun to break down the protective oxide layer, exposing the underlying metal to further attack. The third specimen, exposed to a 5% HCl solution, exhibits severe pitting corrosion with numerous deep cavities and widespread surface deterioration. The extent of material loss is significantly greater compared to the 2.5% corroded specimen, demonstrating the accelerated nature of corrosion at higher acid concentrations. The pits have grown deeper and more interconnected, forming an irregular and highly degraded surface morphology. This aggressive corrosion behavior can be attributed to the enhanced reactivity of chloride ions, which not only disrupt the passive oxide layer but also penetrate deeper into the material, exacerbating localized attack. Additionally, the extensive roughening and material dissolution observed in this specimen suggest that prolonged exposure to high-concentration HCl solutions could further compromise the structural integrity of steel components.

Overall, the SEM analysis confirms that hydrochloric acid exposure leads to progressive and localized deterioration, with the severity of pitting corrosion increasing as the acid concentration rises. These findings emphasize the importance of understanding the relationship between corrosion mechanisms and environmental conditions, as such degradation can significantly impact the durability and performance of steel structures in aggressive chemical environments. The observed corrosion patterns also highlight the necessity for protective measures, such as coatings or inhibitors, to mitigate the detrimental effects of acid-induced material loss in industrial applications.

### 3.4 Evaluation of Theoretical Formulations from the Literature

The collapse buckling values obtained from the experimental study were compared with theoretical formulations derived from the works of Ross^42^ and Jawad,^43^ as reported in the literature (Table 4).

**Table 4:** Theoretical Formulations Obtained from the Literature

	proposed formula	equation number	equation parameters
Ross^42^		(1)	*P*_c_: buckling load; *E*: Young’s modulus; *t*: wall thickness; *r*: radius; *h*: height
Jawad^43^		(2)	

**Figure 10:**
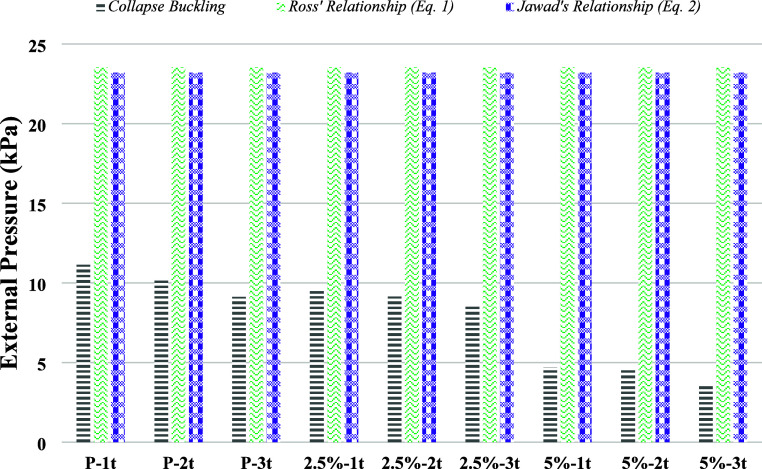
Comparison of theoretical and experimental buckling values

The comparison revealed that the values predicted by both eq 1 and eq 2 were consistently higher than those obtained experimentally. This discrepancy can be attributed to the limitations of the theoretical models, as they do not account for critical factors such as the effects of corrosion, which is commonly encountered in real-world applications, or the presence of imperfections. These omissions highlight the inadequacy of these formulations in accurately representing the buckling behavior of cylindrical specimens under practical conditions. The comparison of theoretical and experimentally obtained buckling values is presented in Table 5 and Figure 10.

**Table 5:** Comparison of Theoretical and Experimental Buckling Values

specimen	collapse buckling (kPa)	Ross’ relationship^42^ (kPa)	error (%)	Jawad’s relationship^43^ (kPa)	error (%)
P-1t	11.14	23.53	52.6	23.20	52.0
P-2t	10.13		56.9		56.3
P-3t	9.12		61.3		60.7
2.5%-1t	9.48		59.7		59.1
2.5%-2t	9.14		61.2		60.6
2.5%-3t	8.62		63.4		62.8
5%-1t	4.67		80.2		79.9
5%-2t	4.54		80.7		80.4
5%-3t	3.65		84.5		84.3

### 3.5 Deformations in Cylindrical Specimens

**Figure 11:**
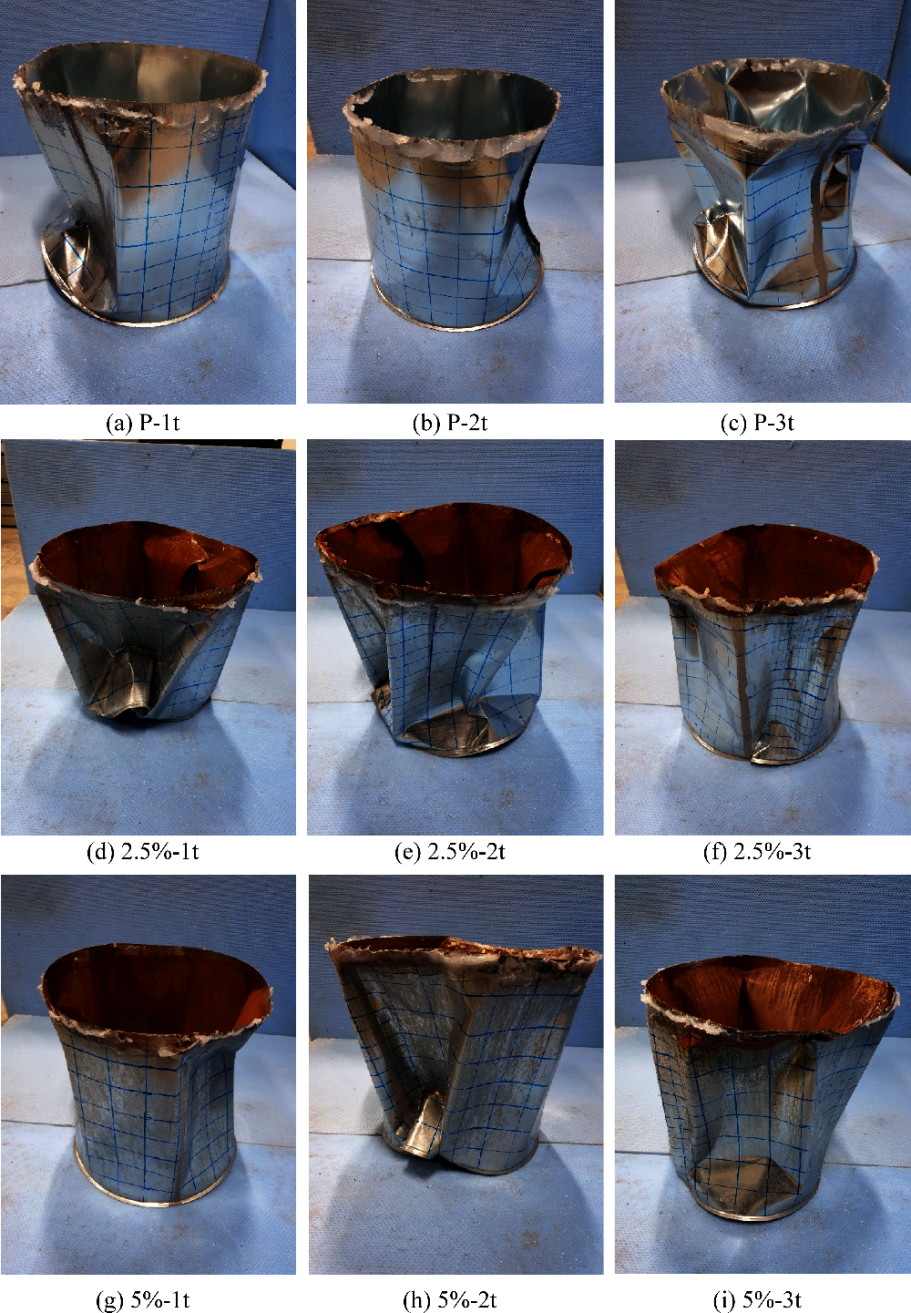
Final forms of experimental samples.

**Figure 12:**
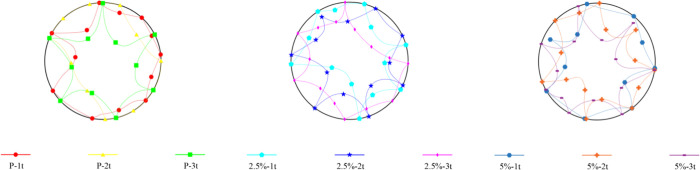
Radial deformations of cylindrical shells.

Buckling due to external pressure loading was observed in the cylindrical specimens. The number of buckling waves varied across the specimens, with noncorroded specimens generally exhibiting fewer waves compared to corroded ones. For instance, noncorroded specimens had wave counts between 2 and 7, while corroded specimens exhibited higher wave counts, ranging between 6 and 7, reflecting the influence of corrosion on the buckling pattern. The presence of 6–7 waves in the noncorroded specimens indicates the potential existence of undetectable imperfections in the cylinders. During deformation, the buckling waves predominantly formed in the regions where the cylindrical shell sections were joined and around the dented areas, highlighting these as critical zones for instability. The final deformed shapes of the cylindrical specimens are presented in Figure 11, while the formation of buckling waves is shown in Figure 12.

### 3.6 Comparison of Experimental Buckling Capacity with Previous Study

Maali et al.^6^ investigated the buckling capacity of cylindrical specimens with the same dimensions as those used in this study. Their work focused on dent-free, noncorroded specimens and specimens corroded using 2.5% and 5% hydrochloric acid (P, 2.5%-P and 5%-P). A comparison of the buckling capacity of these specimens with the dented, noncorroded, and 2.5% and 5% hydrochloric acid-corroded specimens from this study is presented in Table 6.

**Table 6:** Comparison of Buckling Capacity for Dented and Dent-Free Specimens

current study		study of Maali et al.^6^	
specimen	**collapse buckling (kPa)**	**specimen**	**collapse buckling (kPa)**
P-1t	11.14	P	12.38
P-2t	10.13		
P-3t	9.12		
2.5%-1t	9.48	2.5%-P	10.66
2.5%-2t	9.14		
2.5%-3t	8.62		
5%-1t	4.67	5%-P	5.33
5%-2t	4.54		
5%-3t	3.65		

For all conditions, the collapse buckling values in the study from the literature are consistently higher than those in the current study. The comparison shows that the presence of dents in the specimens leads to a reduction in collapse buckling capacity across all conditions. For noncorroded specimens, the dented specimen from the current study (P-1t) exhibits a 10% reduction in collapse buckling capacity compared to the dent-free specimen in Maali et al.’s study (from 12.38 to 11.14 kPa). In the 2.5% hydrochloric acid-corroded condition, the dented specimen (2.5%-1t) shows an approximate 11% reduction compared to the dent-free specimen (from 10.66 to 9.48 kPa). Similarly, for the 5% corroded condition, the dented specimen (5%-1t) experiences a 12% reduction in collapse buckling capacity compared to the dent-free specimen (from 5.33 to 4.67 kPa). These reductions highlight the significant detrimental effect of dents on the structural performance of cylindrical specimens.

To address the experimental repeatability, a consistent trend in collapse buckling capacity reductions has been identified. Across all test conditions (noncorroded, 2.5% HCl, and 5% HCl), the dented specimens exhibit reductions of approximately 10–12% compared to dent-free specimens. This consistent observation across noncorroded and corroded conditions demonstrates the reliability of the experimental procedure and confirms the validity of the measured results.

## 4 Conclusions

This study investigated the buckling behavior of cylindrical specimens with dents under external pressure loading. A total of nine specimens were tested, including noncorroded and corroded specimens exposed to 2.5% and 5% hydrochloric acid solutions. The specimens included three different dent thicknesses (1t, 2t, and 3t) for each corrosion level, allowing a comprehensive analysis of dent and corrosion effects on buckling capacity. The initial imperfections measured in the specimens were in the range of 2% to 3%, providing a realistic representation of real-world manufacturing and operational conditions. The key findings of the study are as follows:

- The weight loss resulting from corrosion ranged between 6.7% and 7.1% for specimens exposed to the 2.5% hydrochloric acid solution and between 9.2% and 9.8% for specimens exposed to the 5% solution.
- SEM analysis confirmed the progressive deterioration of steel specimens due to hydrochloric acid exposure, with increasing acid concentration leading to more severe pitting corrosion, deeper cavities, and surface roughening, ultimately compromising material integrity and buckling performance.
- The overall buckling load-to-initial buckling load ratio ranged from 1.04 to 4.74, while the collapse load-to-overall buckling load ratio varied between 1.04 and 2.43, reflecting the combined effects of corrosion and dents on the specimens’ buckling performance.
- The total number of buckling waves observed during the experiments varied with corrosion levels. For noncorroded specimens, the number of waves ranged between 2 and 7, for 2.5% corroded specimens, between 6 and 7, and for 5% corroded specimens, between 6 and 7.
- Corrosion significantly reduced the buckling capacity of the specimens. When compared to noncorroded specimens, 2.5% corrosion caused a reduction of up to 11%, while 5% corrosion led to reductions of up to 12% in collapse buckling capacity.
- When compared with theoretical formulations, it was observed that both formulations consistently overestimated the buckling capacities. This discrepancy was attributed to the theoretical models not accounting for real-world effects, such as corrosion and imperfections, which were significant factors in the current study.
- The results of this study have practical implications in various engineering applications. The insights gained from this research can aid in the design, maintenance, and safety assessment of thin-walled cylindrical steel tanks used in industries such as oil and gas storage, water and energy systems, and offshore structures. The ability to quantify the effects of dents and corrosion on buckling capacity provides engineers with valuable data for developing more accurate predictive models and designing structures with enhanced durability under combined mechanical and chemical stressors. These findings also highlight the importance of considering real-world imperfections and corrosion in the development of safety guidelines and codes for thin-walled structures.
